# The Effect of Artificial Oocyte Activation on Blastocyst Development in Patients With Low Blastocyst Rates: A Retrospective Cohort Study

**DOI:** 10.7759/cureus.90557

**Published:** 2025-08-20

**Authors:** Feras Sendy, Robert Hemmings, Isaac-Jacques Kadoch, Wael Jamal, Simon Phillips

**Affiliations:** 1 Obstetrics and Gynecology, King Fahad Medical City, Riyadh, SAU; 2 Obstetrics and Gynecology, University of Montreal Health Centre (CHUM), Montreal, CAN; 3 Reproductive Endocrinology and Infertility, Clinique Ovo, Montreal, CAN

**Keywords:** blastocyst, embryo transfer, fertilization, oocyte activation, pregnancy

## Abstract

Introduction and aim: Physiological oocyte activation requires a synergy between the oocyte and sperm to release calcium (Ca^2+^) through oscillations. The absence of such synergy between the oocyte and sperm leads to a negative impact on oocyte activation. Artificial oocyte activation (AOA) can be performed by mechanical, chemical, or electrical approaches. Studies have shown that AOA is helpful in cases of fertilization failure or low fertilization rate, especially in couples with globozoospermia. However, mixed opinions are present on the effect of AOA on blastocyst rate. Thus, this study aimed to investigate the effect of AOA on blastocyst rate in patients with poor or no blastocyst development on their previous In Vitro Fertilization-Intracytoplasmic Sperm Injection (IVF-ICSI) attempt.

Methods: This retrospective cohort single-center study compared intracytoplasmic sperm injection (ICSI) AOA cycles with previous conventional ICSI cycles, and conventional ICSI without AOA cycles with previous conventional ICSI cycles in couples with failed or low blastocyst rates (<30%) in the original ICSI cycle. In total, 54 couples with suboptimal blastocyst development between January 2018 and October 2023 were included in the study.

Twenty-two couples underwent an ICSI-AOA cycle consisting of calcium ionophore (GM508-CultActive) exposure on their second cycle, and 32 couples underwent conventional ICSI without an AOA cycle on their second cycle. The primary outcome measured was blastocyst rate, and secondary outcomes were the percentage of patients without usable embryos, oocyte maturation, fertilization, and pregnancy rates.

Results: We compared 22 AOA cycles to previous conventional ICSI cycles in the same patients and 32 conventional ICSI cycles without AOA to previous conventional ICSI cycles in the same patients. After AOA, the blastocyst rate was not significantly higher than the control group (48% versus 29%, p=0.19). Conversely, the blastocyst rate was significantly higher in the conventional ICSI without AOA cycles than in the control group (48% versus 24%, p=0.04). The fertilization rate and the percentage of patients without usable embryos were not statistically significant between the first and second cycles in both groups.

Conclusion: The literature still lacks strong evidence for AOA overcoming impaired embryonic development. Therefore, AOA remains reserved for couples with a failed or low fertilization history to improve fertilization results. Optimal laboratory conditions and ovarian stimulation modifications without AOA may improve blastocyst rates. Further research is needed to validate our findings due to the presence of confounding factors, small sample size, and retrospective design of the study.

## Introduction

In the fertility field, 7% of all births have been achieved by assisted reproductive technologies (ART) [1]. Intracytoplasmic sperm injection (ICSI) is a technique used in cases of male infertility, following the failure of in vitro fertilization (IVF), resulting in successful fertilization in 70% of cases [1]. However, certain infertile patients cannot achieve adequate fertilization due to the absence of synergy between the sperm and oocyte to release calcium (Ca^2+^) in the form of oscillations [1]. Globozoospermia is one of the causes that leads to an absence of such synergy due to the abnormal formation or absence of the sperm acrosome [2]. Moreover, globozoospermia impacts oocyte activation due to phospholipase C zeta (PLCζ) abnormal expression, localization, and protein structure [3,4].

Artificial oocyte activation (AOA) can be carried out by mechanical, chemical, or electrical means [5]. Mechanical manipulation of the injecting pipette during ICSI is the least invasive and least effective method [6]. Electrical methodology, by direct voltage, allows extracellular Ca^2+^ entry; however, it has a high oocyte degeneration rate [7]. Thus, using chemical components such as calcimycin or ionomycin for AOA became the most common method [5]. Calcimycin or ionomycin has been linked with success in improving the fertilization rate [8,9]. The European Society of Human and Reproduction (ESHRE) recommends AOA in case of fertilization failure, low fertilization (<30%), or globozoospermia [10]. AOA has been proposed as a possible treatment for embryo development after ICSI [11-13]. Nevertheless, studies have presented mixed opinions concerning increases in the blastocyst and live birth rates [11-13]. Furthermore, each study had different inclusion criteria, study design, and Ca^2+^ ionophore use during AOA.

The primary objective of the present study was to investigate whether AOA using ionomycin can improve the blastocyst rate in patients with poor or no blastocyst development in a previous IVF-ICSI cycle. Secondary objectives included evaluating the percentage of patients without usable embryos, as well as fertilization and pregnancy rates.

This study was previously published as a preprint on the medRxiv server on June 30, 2024 (doi.org/10.1101/2024.06.28.24309669).

## Materials and methods

Study design, setting, and participants

A retrospective cohort single-center study was performed between January 2018 and October 2023, including 54 couples with a history of poor blastocyst development (<30%) in a previous ovarian stimulation cycle. Group 1 consists of 22 patients who underwent an ICSI-AOA cycle following a failed conventional ICSI cycle. Group 2, 32 patients, underwent a second conventional ICSI cycle without AOA following a failed conventional ICSI cycle. Group 2 represents a control group since failed cycles are presented to a multidisciplinary medical council at our clinic to assess options to improve outcomes on a second cycle. Modifications to the ovarian stimulation protocol, including changes of gonadotropins, use of adjuvants, or ovulation trigger medications, are often prescribed. Comparing any improvements seen in the AOA group to those seen in this control group reduces the impact of other modifications to treatment carried out in addition to the AOA. Of the 54 couples included, 22 underwent an ICSI-AOA cycle consisting of calcium ionophore (GM508 CultActive) exposure, and 32 couples underwent conventional ICSI without an AOA cycle. The time between the first and second stimulation cycles ranged between 15 days and 4 years. The mean maternal age ranged between 35 and 36 years of age. Couples were diagnosed with male factor infertility, low ovarian reserve, unexplained female infertility, polycystic ovarian syndrome (PCOS), and genetic disease.

Eligibility criteria

The inclusion criteria were patients with poor or no blastocyst development (<30%) in their first stimulation cycle with conventional ICSI, presence of comparable second stimulation cycles, including either a conventional ICSI cycle or an ICSI-AOA. Both stimulation cycles were performed at our fertility center. No restrictions were applied to the underlying cause of infertility, patients' demographics, or time intervals between the first and second stimulation cycles.

**Figure 1:**
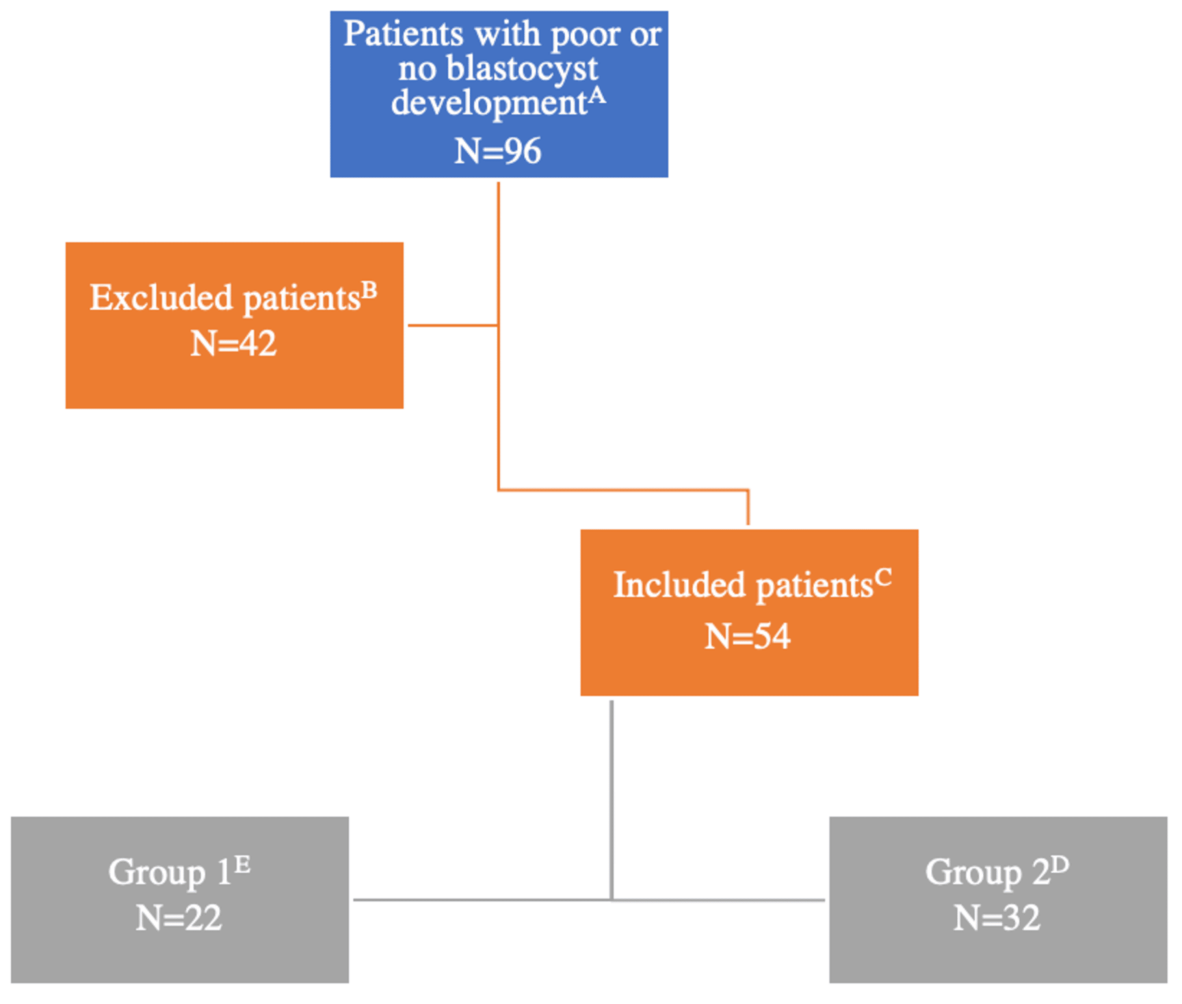
Flowchart explaining the number of included and excluded couples. (A) Patients with stimulation cycles that resulted in poor or no blastocysts (<30%). (B) Patients with absent comparable cycles (without a previous conventional ICSI cycle, second conventional ICSI cycle, or second ICSI-AOA). (C) Patients with comparable cycles. (D) Patients with a first conventional ICSI cycle and a second ICSI-AOA cycle. (E) Patients with a first conventional ICSI cycle and a second conventional ICSI cycle without AOA. ICSI: intracytoplasmic sperm injection; AOA: artificial oocyte activation

The exclusion criteria were patients with good blastocyst development (≥30%) in their first stimulation cycle with conventional ICSI, absence of comparable second stimulation cycles, including either a conventional ICSI cycle or ICSI-AOA. The first or second stimulation cycle is performed in a different fertility center. The flow chart illustrates the included and excluded cases (Figure 1).

ICSI and ICSI-AOA procedures

Female partners were mainly stimulated using an antagonist protocol. Some cases were stimulated using a short agonist protocol. Ovulation was triggered by administering human chorionic gonadotropin (hCG), gonadotropin-releasing hormone agonist (GnRH agonist), or both. At 36-h post-ovulation trigger administration, oocytes were aspirated from the follicles in the ovaries by transvaginal ultrasound-guided puncture in Global Total medium (Trumbull, CT: CooperSurgical). Oocyte denudation was carried out using Cumulase (Trumbull, CT: CooperSurgical), and oocytes were assessed for maturity before ICSI. Sperm selection was performed using density gradient centrifugation or Zymot (Trumbull, CT: CooperSurgical)​​​​​, and fresh or frozen-warmed sperm was used. Only motile spermatozoa were immobilized before injection into mature oocytes. For AOA, oocytes were exposed to GM508 Cultactive (Sierksdorf, Germany: Gynemed GmbH & Co. KG) for 15 minutes post-ICSI, washed through Global Total with HEPES, and cultured in Global Total[14,15]. For both ICSI and ICSI-AOA cycles, embryo culture was performed in Global Total, and embryo transfer was performed fresh on day five or in a frozen embryo transfer cycle using a blastocyst. Gardner's blastocyst grading system was used for the blastocysts [16].

Outcome measures

Patient outcomes include anti-Müllerian hormone (AMH), maturation rate, fertilization rate, the number of two pronuclei, blastocyst rate, blastocyst number, pregnancy rate, and the percentage of patients without embryos. Maturation rate was defined as the number of mature oocytes divided by the number of follicles ≥14 mm. Fertilization rate was defined as the number of pronuclei (2PN) divided by the number of injected mature oocytes. 2PN was defined as the number of fertilized oocytes with two pronuclei (one from the sperm and one from the oocyte). Blastocyst rate was defined as the number of blastocysts divided by the number of 2PN. Pregnancy rate was defined as the number of pregnancies by a positive human chorionic gonadotropin (HCG+) divided by the number of transfers. The percentage of patients without usable embryos was defined as patients without a day five blastocyst.

Statistics and ethical approval

Statistical analysis was performed using SPSS version 26 (Armonk, NY: IBM Corp.). Following assessment of the normal distribution for continuous independent variables, means were expressed with standard deviations and compared by paired t-test, ANOVA, or linear regression. Dependent binary variables, including MII/oocytes (metaphase II; maturation), fertilization (2PN/MII), blastocyst development (blastocyst/2PN), and pregnancy rate (HCG+/transfer), were compared between the first and second cycles in the two groups by z-test or logistic regression. An alpha error of 0.05 was considered significant. The null hypothesis was rejected with a p-value of less than 0.05. This retrospective study was approved by Veritas IRB (#2024-3465-17162-3; dated February 23, 2024). Informed consent was waived due to the retrospective design and the use of de-identified data.

## Results

For group 1, the blastocyst rate was not significantly higher with AOA than conventional ICSI without AOA (48% versus 29%, p=0.19). However, the pregnancy rate was significantly higher with AOA than conventional ICSI without AOA (45% versus 13%, p=0.01). Fertilization and maturation rates were not statistically significant. The percentage of patients without usable embryos was not significantly lower with AOA compared to the conventional ICSI cycle without AOA. All the results for this group are demonstrated in Table 1.

**Table 1:** Results of group 1 patients (second stimulation cycle with ICSI-AOA following a previous conventional ICSI cycle without AOA). *P-value of <0.05 was statistically significant. AMH: anti-Mullerian hormone; ICSI: intracytoplasmic sperm injection; AOA: artificial oocyte activation The maturation rate was calculated as the number of mature oocytes divided by the number of follicles measuring ≥14 mm. The fertilization rate was defined as the number of pronuclei (2PN) divided by the number of injected mature oocytes. 2PN referred to the number of fertilized oocytes with two pronuclei, one from the sperm and one from the oocyte. The blastocyst rate was calculated as the number of blastocysts divided by the number of 2PN. The percentage of patients without usable embryos was defined as those who did not have a day 5 blastocyst. A usable embryo was defined as a day 5 blastocyst. The pregnancy rate was calculated as the number of pregnancies (positive human chorionic gonadotropin) divided by the number of transfers. The z-test was performed for values expressed as percentages, and the t-test was performed for continuous variables expressed as means.

Outcome parameters	Cycle 1: conventional ICSI (N=22)	Cycle 2: ICSI -AOA (N=22)	p-Value	Statistical test (value)
AMH (ng/mL)	3.4±3.2	3.3±3.2	0.91	t-test (-0.10)
Maturation rate (n)	11±7.3	12±10	0.70	t-test (0.23)
Fertilization rate (%)	53%	60%	0.34	z-test (-0.46)
2PN (n)	6.2±5.9	7.3±8.1	0.60	t-test (0.51)
Blastocyst number (n)	1.8±2.5	3.5±3.0	0.04^*^	t-test (2.04)
Blastocyst rate (%)	29%	48%	0.19	z-test (-1.29)
Percentage of patients without usable embryos (%)	54%	27%	0.06	z-test (1.8)
Pregnancy rate, n (%)	3/22 (13%)	10/22 (45%)	0.01^*^	z-test (-2.33)

In group 2, the blastocyst rate was significantly higher with conventional ICSI without AOA than with previous conventional ICSI (48% versus 24%, p=0.04). The pregnancy rate was significantly higher with conventional ICSI without AOA than with previous conventional ICSI (53% versus 28%, p=0.04). However, the maturation and fertilization rates were not statistically significant. The percentage of patients without usable embryos was not significantly lower with the second conventional ICSI cycle than with their previous conventional ICSI cycle. The outcomes for this group are demonstrated in Table 2. Comparative analysis of pregnancy, blastocyst, and fertilization between group 1 and group 2 in the second cycle is summarized in Table 3.

**Table 2:** Results of group 2 (second stimulation cycle with conventional ICSI without AOA following a previous conventional ICSI cycle). *P-value of <0.05 was statistically significant. AMH: anti-Mullerian hormone; ICSI: intracytoplasmic sperm injection; AOA: artificial oocyte activation The maturation rate was defined as the number of mature oocytes divided by the number of follicles measuring ≥14 mm. The fertilization rate was calculated as the number of pronuclei (2PN) divided by the number of injected mature oocytes. 2PN refers to the number of fertilized oocytes containing two pronuclei, one from the sperm and one from the oocyte. The blastocyst rate was defined as the number of blastocysts divided by the number of 2PN. The percentage of patients without usable embryos was defined as those without a day 5 blastocyst. A usable embryo was defined as a day 5 blastocyst. The pregnancy rate was calculated as the number of pregnancies (positive human chorionic gonadotropin) divided by the number of transfers. The z-test was performed for values reported as percentages, and the t-test was performed for continuous variables reported as means.

Outcome parameters	Cycle 1: conventional ICSI (N=32)	Cycle 2: conventional ICSI (N=32)	p-Value	Statistical test (value)
AMH (ng/mL)	3.1±2.6	3.0±2.5	0.87	t-test (0.63)
Maturation rate (n)	7±4.6	8±5	0.40	t-test (0.82)
Fertilization rate (%)	72%	68%	0.72	z-test (0.34)
2PN (n)	4.9±3.6	5.9±3.7	0.27	t-test (1.09)
Blastocyst number (n)	1.2±1.8	2.8±2.3	0.001^*^	t-test (3.09)
Blastocyst rate (%)	24%	48%	0.04^*^	z-test (-2.0)
Percentage of patients without usable embryos (%)	43%	12%	0.005^*^	z-test (2.7)
Pregnancy rate, n (%)	8/28 (28%)	15/28 (53%)	0.04^*^	z-test (-2.0)

**Table 3:** Results from the second cycles of group 2 and group 1. *P-value of <0.05 was statistically significant. ICSI: intracytoplasmic sperm injection; AOA: artificial oocyte activation Group 2 included patients who underwent a second stimulation cycle with conventional ICSI without AOA, following a previous conventional ICSI cycle. Group 1 included patients who underwent a second stimulation cycle with ICSI-AOA, following a previous conventional ICSI cycle without AOA. The z-test was performed for the variables.

Outcome parameters	Group 2 (N=32)	Groupe 1 (N=22)	p-Value	Statistical test (value)
Percentage of fertilization (%)	68%	60%	0.54	z-test (0.60)
Percentage of blastocyst (%)	48%	48%	1.0	z-test (-0.88)
Percentage of pregnancy (%)	53%	45%	0.54	z-test (-0.66)

## Discussion

In this study, we showed that AOA treatment neither improved the blastocyst rate nor the pregnancy rate in our patient cohort with previous poor blastocyst rates (<30% blastocyst formation). The findings were not influenced by fertilization, as both cycles had comparable rates. Although the cycle-to-cycle duration effect was not measured, we believe that the duration between cycles does not seem to impact results, given that no significant changes occurred in the laboratory culture system during the period under evaluation. Furthermore, any generalized improvements would have primarily affected the second cycle of the AOA group, adding more weight to the findings that AOA did not improve the blastocyst development rate.

There is evidence that ICSI-AOA improves fertilization rates, leading to blastocysts in patients with poor fertilization [17-19]. Oocyte activation is impacted by the sperm or oocyte due to the absence of synergy to produce Ca^2+^. As a result, AOA during fertilization helps conquer this issue. Other studies did not find an improved effect [20,21]. AOA has been proposed for embryo development after ICSI, yet the results remain mixed [11-13]. For example, Ebner et al. used GM508 Cult-Active to treat 57 patients with reduced blastocyst rate (≤15%) and found a significant increase in blastocyst and pregnancy rates after AOA [11]. Similarly, our study found a significant increase in pregnancy rate in group 1 (Table 1). However, the blastocyst rate was not statistically significant.

Similarly, results did not find AOA’s advantage in improving blastocyst rate [12,13]. Yin et al. used ionomycin to treat 140 patients with reduced day three embryo rate (≤30%) and found no difference in day three embryo, blastocyst, and pregnancy rates [13]. The inclusion criteria of poor blastocyst rate (<30%) in our study was different from Yin et al. and Ebner et al. who used cutoffs of poor cleavage stage (≤30%) and poor blastocyst stage (≤15%) Mateizel et al. used calcimycin to treat 15 patients with poor blastocyst rate and found no difference in blastocyst rate [11-13]. Both studies used different Ca^2+^ ionophores (calcimycin versus ionomycin) and embryo stages (blastocyst versus cleavage) [12,13]. Nonetheless, they used sibling oocytes as a control group and found similar outcomes. Moreover, Ebner et al. compared ICSI-AOA to the previous ICSI cycle with a poor blastocyst rate, a similar criterion in our study [11].

The lack of Ca^2+^ oscillations during fertilization is one of the reasons for fertilization failure. A study found that AOA may develop higher activation using ionomycin rather than calcimycin [22]. Some patients with failed ICSI-AOA by calcimycin may be rescued by ionomycin [23]. Thus, much higher stimuli are required for some patients than others [24]. We could not examine the effect of using different ionophores or sibling oocytes. However, our study compared group 1 (Table 1), group 2 (Table 2), and both groups but did not find a significant impact on fertilization rate (Table 3).

Our study did not evaluate the miscarriage or live birth rate. However, the literature provides evidence of AOA’s effectiveness and safety when used in adequate conditions (fertilization failure or low fertilization). A meta-analysis of 14 studies found that AOA increased live birth and pregnancy rates with no significant effect on miscarriage rate [19]. Furthermore, globozoospermia is an indication of AOA by ionomycin than calcimycin (30% versus 11.8%) [9]. Chromosomal abnormalities, gene expression, or morphokinetics due to Ca^2+^ ionophores are unlikely due to the inability of Ca^2+^ to enter the oocyte [25,26]. Moreover, various studies reported the absence of any link between AOA and birth defects [27-29]. Likewise, cognition, language, and motor skills were normal in children [30].

The stimulation protocol and dosage choice depend on the patient’s age, weight, ovarian reserve, and response to previous stimulation. The use of antagonist stimulation protocol resulted in a comparable ongoing pregnancy rate to agonist stimulation protocol for poor responders in a meta-analysis involving 50 studies [31]. A randomized controlled trial looked at the impact of constant versus increased stimulation dosage under antagonist protocol [32]. The number of oocytes retrieved, fertilization, and pregnancy rates were not significantly different. Furthermore, a systematic review of 30 studies compared stimulation with recombinant follicular-stimulating hormone (rFSH) and rFSH with recombinant luteinizing hormone (rLH) [33]. They found that hypo-responders and individuals of advanced age (36-39 years old) might have better oocyte retrieval and implantation rates in stimulation using rFSH and rLH. However, a randomized controlled trial in 240 patients over 35 years old did not find a significant live birth rate in rFSH and rLH compared to rFSH alone [34]. Moreover, a meta-analysis of 22 studies found comparable clinical pregnancy rates yet lower consumption of FSH in poor responders with clomiphene citrate or aromatase inhibitors during stimulation in comparison to ovarian stimulation without the addition of clomiphene citrate or aromatase inhibitors [35]. Ovulation induction using an agonist or HCG showed a comparable number of oocytes retrieved and live birth rate in a randomized controlled trial for 257 women undergoing oocyte donation [36]. Using double triggers (agonist and HCG) for ovulation induction has been advocated for increasing oocyte maturation [37]. Most of our patients had an antagonist stimulation protocol with or without aromatase inhibitors, and some of them had an ovulation trigger using the agonist alone or in combination with HCG, depending on the risk of ovarian hyperstimulation syndrome. Others had short agonist stimulation due to a change in previous stimulation, a poor response, or other reasons. The results of the second stimulation cycle in conventional ICSI and ICSI-AOA have improved compared to the first. The improvement could be attributed to the change of stimulation protocol, the inadequate dosage of stimulation in the first cycle, or the absence of a double trigger in some cases.

A recent retrospective study involving 802 patients undergoing a second ovarian stimulation with the same dosage of medications found that 50% of the patients had a different response [38]. In the study inclusion criteria, patients had two or more stimulation cycles within six months. Our study did not have a limited time interval, yet variation of results between our first and second stimulation cycles were found as well. The difference in pregnancy rate in the same patients undergoing a second stimulation cycle in our study could be due to individual intervariability. The absence of a clinical pregnancy rate in our study could be an arguable limitation, as biochemical pregnancies reported in our study may not progress toward a clinical pregnancy.

Our data show that AOA is not indicated for patients with a history of poor blastocyst rate after ICSI (<30%), as it probably results from other factors not related to Ca^2+^. Despite our low sample size, we have shown that AOA does not improve blastocyst rate since group 2 patients improved on their second conventional ICSI cycle without this intervention being applied. This technique should be reserved for patients with failed or low fertilization [10]. Optimizing laboratory conditions and ovarian stimulation protocols may improve blastocyst rates for such patients.

Limitations

Our strict inclusion criteria, requiring at least one previous ICSI cycle with a failed or low blastocyst development rate, permitted an ideal comparison of the same patient groups. However, this criterion limited the sample size of the study, which may have increased the risk of bias. The lack of blinding and the presence of various confounding factors, including male factor infertility, low ovarian reserve, unexplained female infertility, polycystic ovarian syndrome (PCOS), and genetic disease, may have affected our results. Furthermore, we included both male and female factors in the study, which could be probable reasons for failed ICSI (poor blastocyst rates <30%), and may also increase the risk of bias.

## Conclusions

The literature lacks strong evidence for AOA overcoming impaired embryonic development. This study suggests that the addition of AOA in patients with a history of low blastocyst rate does not lead to a higher blastocyst formation rate compared to repeated IVF with ICS cycle. Therefore, AOA should be reserved for couples with a failed or low fertilization history to improve fertilization results. The outcomes of the study should not be overgeneralized due to the small sample size and potential confounding factors, including variable stimulation protocols and male/female infertility factors. Further studies are needed for statistical validation and subgroup analysis to support our findings.

## References

[1] Nasr-Esfahani MH, Deemeh MR, Tavalaee M (2010). Artificial oocyte activation and intracytoplasmic sperm injection. Fertil Steril.

[2] Dam AH, Feenstra I, Westphal JR, Ramos L, van Golde RJ, Kremer JA (2007). Globozoospermia revisited. Hum Reprod Update.

[3] Heytens E, Parrington J, Coward K (2009). Reduced amounts and abnormal forms of phospholipase C zeta (PLCζ) in spermatozoa from infertile men. Hum Reprod.

[4] Yoon SY, Jellerette T, Salicioni AM (2008). Human sperm devoid of PLC, zeta 1 fail to induce Ca2+ release and are unable to initiate the first step of embryo development. J Clin Invest.

[5] Kashir J, Ganesh D, Jones C, Coward K (2022). Oocyte activation deficiency and assisted oocyte activation: mechanisms, obstacles and prospects for clinical application. Hum Reprod Open.

[6] Ebner T, Moser M, Sommergruber M, Jesacher K, Tews G (2004). Complete oocyte activation failure after ICSI can be overcome by a modified injection technique. Hum Reprod.

[7] Yanagida K, Katayose H, Yazawa H, Kimura Y, Sato A, Yanagimachi H, Yanagimachi R (1999). Successful fertilization and pregnancy following ICSI and electrical oocyte activation. Hum Reprod.

[8] Ebner T, Köster M, Shebl O, Moser M, Van der Ven H, Tews G, Montag M (2012). Application of a ready-to-use calcium ionophore increases rates of fertilization and pregnancy in severe male factor infertility. Fertil Steril.

[9] Nikiforaki D, Meerschaut FV, de Roo C, Lu Y, Ferrer-Buitrago M, de Sutter P, Heindryckx B (2016). Effect of two assisted oocyte activation protocols used to overcome fertilization failure on the activation potential and calcium releasing pattern. Fertil Steril.

[10] Lundin K, Bentzen JG, Bozdag G (2023). Good practice recommendations on add-ons in reproductive medicine. Hum Reprod.

[11] Ebner T, Oppelt P, Wöber M (2015). Treatment with Ca2+ ionophore improves embryo development and outcome in cases with previous developmental problems: a prospective multicenter study. Hum Reprod.

[12] Mateizel I, Santos-Ribeiro S, Segers I, Wouters K, Mackens S, Verheyen G (2022). Effect of A23187 ionophore treatment on human blastocyst development - a sibling oocyte study. J Assist Reprod Genet.

[13] Yin M, Li M, Li W (2022). Efficacy of artificial oocyte activation in patients with embryo developmental problems: a sibling oocyte control study. Arch Gynecol Obstet.

[14] Murugesu S, Saso S, Jones BP (2017). Does the use of calcium ionophore during artificial oocyte activation demonstrate an effect on pregnancy rate? A meta-analysis. Fertil Steril.

[15] Ebner T, Montag M, Montag M (2015). Live birth after artificial oocyte activation using a ready-to-use ionophore: a prospective multicentre study. Reprod Biomed Online.

[16] Gardner DK, Schoolcraft WB (1999). Culture and transfer of human blastocysts. Curr Opin Obstet Gynecol.

[17] Bonte D, Ferrer-Buitrago M, Dhaenens L (2019). Assisted oocyte activation significantly increases fertilization and pregnancy outcome in patients with low and total failed fertilization after intracytoplasmic sperm injection: a 17-year retrospective study. Fertil Steril.

[18] Li J, Zheng X, Lian Y (2019). Artificial oocyte activation improves cycles with prospects of ICSI fertilization failure: a sibling oocyte control study. Reprod Biomed Online.

[19] Shan Y, Zhao H, Zhao D, Wang J, Cui Y, Bao H (2021). Assisted oocyte activation with calcium ionophore improves pregnancy outcomes and offspring safety in infertile patients: a systematic review and meta-analysis. Front Physiol.

[20] Sang Q, Li B, Kuang Y (2018). Homozygous mutations in WEE2 cause fertilization failure and female infertility. Am J Hum Genet.

[21] Barberán AC, Boel A, Meerschaut FV, Stoop D, Heindryckx B (2020). Diagnosis and treatment of male infertility-related fertilization failure. J Clin Med.

[22] Jia L, Chen P, Su W (2023). Artificial oocyte activation with ionomycin compared with A23187 among patients at risk of failed or impaired fertilization. Reprod Biomed Online.

[23] Ebner T, Shebl O, Oppelt P (2021). First live births after application of a ready-to-use ionomycin in cases of failed artificial oocyte activation (AOA) using calcimycin. Fertil Steril.

[24] Ebner T, Shebl O (2022). Effect of A23187 ionophore treatment on human blastocyst development - a sibling oocyte study. J Assist Reprod Genet.

[25] Capalbo A, Ottolini CS, Griffin DK, Ubaldi FM, Handyside AH, Rienzi L (2016). Artificial oocyte activation with calcium ionophore does not cause a widespread increase in chromosome segregation errors in the second meiotic division of the oocyte. Fertil Steril.

[26] Shebl O, Trautner PS, Enengl S (2021). Ionophore application for artificial oocyte activation and its potential effect on morphokinetics: a sibling oocyte study. J Assist Reprod Genet.

[27] Mateizel I, Verheyen G, Van de Velde H, Tournaye H, Belva F (2018). Obstetric and neonatal outcome following ICSI with assisted oocyte activation by calcium ionophore treatment. J Assist Reprod Genet.

[28] Long R, Wang M, Yang QY, Hu SQ, Zhu LX, Jin L (2020). Risk of birth defects in children conceived by artificial oocyte activation and intracytoplasmic sperm injection: a meta-analysis. Reprod Biol Endocrinol.

[29] Deemeh MR, Tavalaee M, Nasr-Esfahani MH (2015). Health of children born through artificial oocyte activation: a pilot study. Reprod Sci.

[30] Meerschaut FV, D'Haeseleer E, Gysels H (2014). Neonatal and neurodevelopmental outcome of children aged 3-10 years born following assisted oocyte activation. Reprod Biomed Online.

[31] Lambalk CB, Banga FR, Huirne JA (2017). GnRH antagonist versus long agonist protocols in IVF: a systematic review and meta-analysis accounting for patient type. Hum Reprod Update.

[32] Aboulghar MA, Mansour RT, Serour GI, Al-Inany HG, Amin YM, Aboulghar MM (2004). Increasing the dose of human menopausal gonadotrophins on day of GnRH antagonist administration: randomized controlled trial. Reprod Biomed Online.

[33] Alviggi C, Conforti A, Esteves SC (2018). Recombinant luteinizing hormone supplementation in assisted reproductive technology: a systematic review. Fertil Steril.

[34] Vuong TN, Phung HT, Ho MT (2015). Recombinant follicle-stimulating hormone and recombinant luteinizing hormone versus recombinant follicle-stimulating hormone alone during GnRH antagonist ovarian stimulation in patients aged ≥35 years: a randomized controlled trial. Hum Reprod.

[35] Bechtejew TN, Nadai MN, Nastri CO, Martins WP (2017). Clomiphene citrate and letrozole to reduce follicle-stimulating hormone consumption during ovarian stimulation: systematic review and meta-analysis. Ultrasound Obstet Gynecol.

[36] Galindo A, Bodri D, Guillén JJ, Colodrón M, Vernaeve V, Coll O (2009). Triggering with HCG or GnRH agonist in GnRH antagonist treated oocyte donation cycles: a randomised clinical trial. Gynecol Endocrinol.

[37] Hong YH, Kim SK, Lee JR, Jee BC, Suh CS (2022). Clinical efficacy of dual trigger with human chorionic gonadotropin and a gonadotropin-releasing hormone agonist for women undergoing fertility preservation. Reprod Med Biol.

[38] Hochberg A, Maman E, Polyzos NP (2025). O-287 Inter-cycle intra-patient variability in the retrieved oocyte numbers and ovarian response categories between consecutive IVF cycles with identical gonadotropin type, dose, and ovarian stimulation protocol. Hum Reprod.

